# Homeostasis as the Mechanism of Evolution

**DOI:** 10.3390/biology4030573

**Published:** 2015-09-15

**Authors:** John S. Torday

**Affiliations:** Harbor-UCLA Medical Center, 1224 W. Carson Street, Torrance, CA 90502, USA; E-Mail: jtorday@labiomed.org; Tel.: +1-310-222-8186, Fax: +1-310-222-3887

**Keywords:** evolution, homeostasis, development, phylogeny, epigenetics, diachronic, cell-cell signaling, embryogenesis, scale-free, teleology

## Abstract

Homeostasis is conventionally thought of merely as a synchronic (same time) servo-mechanism that maintains the status quo for organismal physiology. However, when seen from the perspective of developmental physiology, homeostasis is a robust, dynamic, intergenerational, diachronic (across-time) mechanism for the maintenance, perpetuation and modification of physiologic structure and function. The integral relationships generated by cell-cell signaling for the mechanisms of embryogenesis, physiology and repair provide the needed insight to the scale-free universality of the homeostatic principle, offering a novel opportunity for a Systems approach to Biology. Starting with the inception of life itself, *with the advent* of reproduction during meiosis and mitosis, moving forward both ontogenetically and phylogenetically through the evolutionary steps involved in adaptation to an ever-changing environment, Biology and Evolution Theory need no longer default to teleology.

## 1. Introduction

Any systematic approach to Biology and Medicine should ideally be based on ontologic and epistemologic first principles. This has proven to be challenging until now, given the observational and descriptive nature of Biology, which Earnest Rutherford characterized as ‘stamp collecting’ [1]. Seen from a cellular-molecular perspective [2,3,4], homeostasis is the mechanistic fundament of biology, beginning with the protocell [2].

Teleology has been helpful historically in understanding biologic purpose, but harmful in limiting thinking about mechanistic origins, because evolved traits are permutations and combinations of otherwise-purposed historic traits. It is what Jacob had referred to as ‘tinkering’ [5], but should be thought of within physiologic boundaries. For example, there were specific gene duplications and mutations that occurred during the vertebrate water-land transition [3,4]—the duplications of both the Parathyroid Hormone-related Protein (PTHrP) Receptor [6], and the β Adrenergic Receptor [7], and the mutation of the Mineralocorticoid Receptor to form the Glucocorticoid Receptor [8]. All three of these mutations were key to the physiologic changes necessary for land adaptation—skeletal, pulmonary, kidney, skin, and vascular [3,4]. The repurposing of these genes was the aggregate consequence of past conditions, allowing for the informed emergence of future biologic developmental traits.

In order to understand the historic conditions of the past that led up to these events, a novel approach to understanding the processes involved in *eukaryotic* evolution based on the unicellular state of the life cycle as the principal level of selection has been taken [2,3,4]. This view assumes that the cell originated from the primordium through the spontaneous formation of micelles [2], generating a protected space within which chemical catalysis generated chemiosmotic energy [9,10,11], facilitating a reduction in entropy, sustained and perpetuated by homeostatic mechanisms [12]. That perspective subsumes a diachronic, or across-time process for mechanistically interconnecting the past, present and future of the organism [13], rather than the conventional synchronic, quasi-static view of homeostasis as merely maintaining the *status quo* [14]. The principle reason that this point of view has not been put forward previously is because of the wide-spread, pervasive acceptance of teleological thinking in biology [15]. I would like to offer a causal and predictive way of thinking about Biology that would obviate the need for such teleology and dogma.

## 2. Homeostasis is Anything But Static

Traditionally, Biologists have described the flora and fauna, pulling them apart to determine their structure and function [16]. However, something of a revolution occurred during the second half of the 20th century when it was discovered that isolated epithelial cells lost their differentiated phenotype when they were propagated in cell culture [17]. Conversely, providing these epithelial cells with their investing fibroblasts restored their structure and function, explaining, for example, why intact embryonic tissue continued to develop along its normal trajectory in culture [18]. These observations integrated mechanisms of cellular development and homeostasis for the first time. Moreover, the Fetal Origins of Adult Disease, or the Barker Hypothesis, in combination with epigenetic inheritance causally integrated homeostasis between generations. These key observations have led to a mechanistic understanding of evolution based on cell-cell signaling [2,3,4].

Elsewhere [2,3,4], it has been suggested that the entire mechanism of evolution must be seen simultaneously as the organism’s ontogenetic and phylogenetic history. Nowhere is the dichotomy between conventional descriptive biology and mechanistically-dynamic evolution more apparent than in the way we only think of homeostasis as static, like a household thermostat. Homeostasis is constantly oscillating around a set-point, monitoring the cellular environment, always ready to reset itself, but also to provide the reference point for change if necessary for survival in an ever-changing environment. Whereas the perspective that homeostasis is static is based on contemporary descriptive biology, the dynamic perspective is best seen in the field of developmental physiology, particularly when it is truncated in the preterm infant [19,20,21], or reversed, as in chronic diseases [22]. For it is the growth factor signaling mechanisms of development, regeneration and repair that underlie all of these processes, providing a way of seeing the continuum by which structure and function change over the evolutionary course of ontogeny and phylogeny, and attain equipoise to maintain [23], sustain [24] and perpetuate [25] physiologic stability.

An earlier paper [26] discussed a mechanistically-based continuum from ontogeny and phylogeny to homeostasis and regeneration based on the underlying cell-cell interactions that determine lung surfactant biogenesis in service to homeostasis-absent surfactant, the alveoli will collapse due to the effect of surface tension. The merging of ontogeny and phylogeny into one continuous mechanism of lung homeostasis turns out to be a unique insight to the fundamental mechanism of evolution—how homeostasis can act simultaneously as both a stabilizing agent and as the determining mechanism for evolutionary change. Robert G.B. Reid, in his book Evolutionary Theory—the unfinished synthesis [27], pointed out the paradoxical relationship between homeostasis and evolution, though he failed to invoke the needed developmental dimension. Biologists make a systematic error in describing the different phases of the life cycle without considering the mechanistic interrelationships between them, which must logically exist, but they have been siloed and coopted by the various sub-disciplines of Biology. It is this fractious nature of descriptive biology that is hindering our understanding of what evolution actually constitutes.

## 3. The Historic Concept of Homeostasis, from Bernard to Cannon 

Homeostasis is defined as the property of a system in which variables are regulated so that internal conditions remain stable and relatively constant. Examples of homeostasis include the regulation of body temperature, and the balance between acidity and alkalinity. It is a process that maintains the stability of the organism’s internal environment in response to fluctuations in external environmental conditions.

Claude Bernard first described the processes of physiologic control as the *milieu interieur* in his book. An Introduction to the Study of Experimental Medicine [28]. The term homeostasis was later coined by Walter Bradford Cannon in his book Organization for Physiological Homeostasis [29]. Waddington [30] preferred the more dynamic term homeorhesis. Although the term homeostasis was originally used to refer to processes within living organisms, it is frequently applied to autonomous control systems ranging from cruise control to celestial bodies. Homeostasis requires a sensor to detect changes in the condition to be regulated, an effector mechanism that can vary that condition, and a negative feedback connection between the two.

Every living organism depends on maintaining a complex set of interacting metabolic chemical reactions. From the simplest unicellular organisms, to the most complex plants and animals, internal processes operate to keep their conditions within tightly regulated and controlled limits to allow these reactions to proceed. Homeostatic processes act at the level of the cell, the tissue, and the organ, as well as at the level of the organism as a whole, referred to as allostasis [31,32].

All homeostatic control mechanisms have at least three interdependent components for the variable being regulated: The receptor is the sensing component that monitors and responds to changes in the environment. When the receptor senses a stimulus, it signals information to the nucleus, which sets the range at which the variable is maintained. The nucleus determines an appropriate response to the stimulus. The nucleus then sends signals to an effector, which can be other cells, tissues, organs, or other structures that receive signals for homeostasis. After receiving the signal, a change occurs to correct the deviation by depressing or damping it utilizing negative feedback [33].

## 4. Negative Feedback

Negative feedback mechanisms consist of reducing the output or gain of any organ or system back to its normal range of function [34]. A good example is the regulation of blood pressure. Blood vessels can sense the resistance to blood flow when blood pressure increases. The blood vessels act as receptors, relaying the message to the brain. The brain then sends a message to the heart and blood vessels, both of which are effectors. The heart rate will decrease as the blood vessels increase in diameter (vasodilation). This change causes the blood pressure to fall back into its normal range. The opposite happens when blood pressure decreases, causing vasoconstriction.

Another important example is seen when the body is deprived of food. In response, the body will reset the metabolic set-point to a lower value. This allows the body to continue to function at a slower metabolic rate even though the body is starving. Therefore, people depriving themselves of food while trying to lose weight find it easy to shed weight initially, but much harder to lose more thereafter. This is due to the body automatically readjusting itself to a lower metabolic set-point to allow it to survive with its lower supply of energy. Exercise can change this effect by increasing the metabolic demand.

Yet another good example of a negative feedback mechanism is body temperature control. The hypothalamus, which monitors body temperature, is capable of determining even the slightest variations in body temperature. Response to such variation could be stimulation of glands that produce sweat to reduce the temperature, or signaling various muscles to shiver to increase body temperature.

## 5. Homeostatic Imbalance

Many diseases involve disturbances in homeostasis. For example, as the organism ages, the efficiency in the control of systems becomes reduced due to the loss of receptors. The inefficiencies gradually result in an unstable internal environment that increases the risk of illness, leading to the physical changes associated with aging [2].

Certain homeostatic imbalances, such as a high core body temperature, a high concentration of salt in the blood, or a low concentration of oxygen, can generate homeostatic reactions such as warmth, thirst, or breathlessness, which motivate behavior aimed at restoring homeostasis [35].

## 6. The Cart and Horse Diachronic Perspective

*“It is not enough to see the horse pulling a cart past the window as the good working horse it is today; the picture must also include the minute fertilized egg, the embryo in its mother’s womb, and the broken-down old nag it will eventually become.”* Conrad Hal Waddington [30].

There is a fundamental epistemologic (cart and horse) problem in Evolution Theory—the perspective on the life cycle. As Waddington suggests in the quote above, it is necessary to see the entire process of life as a continuum in order to understand the underlying evolutionary principles involved. The first paper published on the cellular approach to vertebrate evolution [26] reduced both lung evolution and physiology to its cellular, mechanistic level, which allowed examination of ontogeny and phylogeny across space and time as one continuous process, i.e. the short- and long-term histories of this organ are, in fact, one and the same. In retrospect, that was an important breakthrough because it demonstrated the fallacy in looking at ontogeny and phylogeny as independent of one another, as would seem to be the case when looked at from the perspective of their present forms. But by looking at the process from its cell-molecular mechanistic basis, one can see it longitudinally as a continuous process of adaptation, largely to atmospheric oxygen, accommodating the metabolic demand for vertebrate evolution.

That conceptual breakthrough has more recently led to reconsideration of Haeckel’s Biogenetic Law that Ontogeny recapitulates Phylogeny in a new light. Perhaps Haeckel overstated the case in his zeal to identify the basis for evolution, but by inferring that the two processes have common properties, he provided an important insight to the mechanism of evolution, begging the question as to what underlies Ontogeny and Phylogeny. Whatever that property is, it has allowed vertebrates to adapt to a changing oxygen environment over eons, somehow mediated through the processes of embryogenesis. The most logical mechanism that transcends such diverse scales of adaptation is homeostasis. And the recapitulation of phylogeny may act to constrain evolutionary changes that are internally consistent with homeostatic control at key stages of embryologic development. 

That, in turn, raises the question as to what homeostasis has evolved in support of? It has been suggested [2] that reduced entropy is the driving force behind evolution, a property of life that requires homeostatic control to be sustained and perpetuated. Reducing evolution to homeostasis offers a fundamental mechanistic insight to the origin and causal nature of this process. It is no longer random mutation and Natural Selection, but adaptation of the internal environment of the organism to the external environment of the physical world in service to homeostasis. Ultimately, Selection facilitates the homeostatically-determined change resulting from the interaction between the organism and the environment.

In their millennial paper on evolution, entitled “Molecular Vitalism” [36] Kirschner, Gerhart and Mitchelson cited Gerhard Fankhauser’s classic paper on the effects of cell size on newt development [37]. Polyploid embryos had fewer but larger cells, which had no effect on tissue or body size. For example, kidney duct size remained unaffected by the reduced number of epithelial cells surrounding it. This finding even flummoxed the great Einstein [38], prompting him to state that “It looks as if the importance of the cell as ruling element of the whole had been overestimated previously. What the real determinant of form and organization is seems obscure”. Fankhauser then questioned what the real determinant of form and organization is. Yet, if one hypothesizes that homeostasis is the underlying selection pressure for solute exchange over the surface area of the kidney duct, the absence of overall structural change now makes sense.

In short, we have been putting the phenotypic-genotypic ‘cart’ before the homeostatic‘horse’ for far too long, allowing our thought processes to be diverted by descriptive, top-down, *a posteriori* biology. We must begin thinking along cellular-molecular lines regarding evolution if we are to make advances in Biology and Medicine, or we will languish as the Alchemists did until Chemistry, the Periodic Table and Quantum Theory finally set us on the road to predictive Physics.

## 7. Homeostatic Regulation as Downward Causation

Downward Causation describes a causal relationship from higher levels of a system to lower-level components of that system. For example, mental events acting to cause physical events. The term was coined in 1974 by the philosopher and social scientist Donald T. Campbell [39].

**Figure 1:**
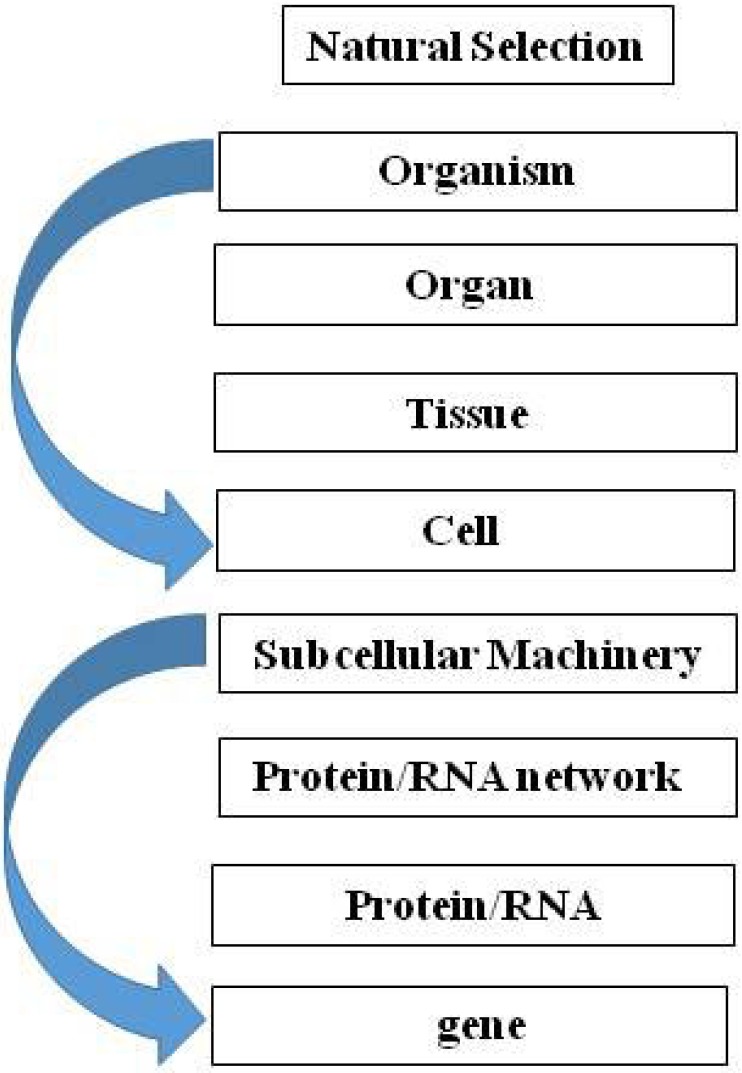
Downward Causation. Teleologically, there is no privileged level of causality in biological systems.

In the paper “A theory of biological relativity: no privileged level of causation” [40], the author makes the case for Downward Causation (Figure 1). He concludes from this approach that there is no privileged level of selection in biological systems. This is a classic example of the systematic error we make in thinking about life forms culminating in their adult state, in contrast to Waddington’s ‘cart and horse’ imagery, which encourages us to think beyond the present circumstance to the continuum of life, including the next generation (the fertilized egg in the mother’s womb) [30]. As a result, for example, Noble [40] concludes that teleologically there is no privileged level of causality in biological systems. However there is, but it can only be seen by running the tape forward from unicellular state to unicellular state over the entire course of the life cycle of the organism. We now know that the epigenetic ‘marks’ acquired during the life of the organism are not all eliminated during meiosis, as had previously been thought [41,42,43], and that those marks are heritable and biologically active [44]. Our laboratory has been studying the effect of maternal nicotine exposure on the transgenerational inheritance of the asthma phenotype [45]. Nicotine induces specific epigenetic changes in both the upper airway of the lung and the gonads of the offspring for at least three generations. This is the first experimental evidence for true epigenetic transgenerational inheritance. These findings beg the question as to the level of selection because newly-acquired epigenetic mutations only affect the offspring, not the adults.

**Figure 2:**
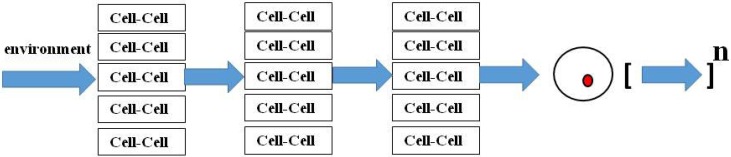
Evolution as Cell-Cell Signaling. Environmental ‘stress’ affects cell-cell communication mechanisms that determine homeostatic control, resulting in genetic ‘mutations’ that modify structure and function evolutionarily.

The Downward Causation perspective requires reassessment of the bias towards the ‘vertical’ approach to the multi-leveled generalization of Darwin’s theory [46], which can be top-down or bottom-up, whereas I have been advocating for a ‘middle-out’ cell-cell signaling approach [2,3,4]. The vertical perspective seems more in line with conventional Darwinian ‘Descent with Modification’ and Natural Selection, whereas the middle-out approach across spatio-temporal ontogeny and phylogeny is more in concert with the effects of environmental forces such as the Sun (above) and gravity (below). Seen as a vectorial product of these forces (Figure 2), evolution would be propelled horizontally from generation to generation, constantly gaining information from the environment in the process.

Perhaps the reason why we go through the life cycle from zygote to zygote is to acquire epigenetically-heritable information from the environment and selectively integrate it into our genome. The ‘filtering’ mechanisms are those of ontogeny and phylogeny, providing both the short-term and long-term ‘histories’ of the organism as a means of monitoring the homeostatic relevance of the acquired mutations. Homeostasis is integral to morphogenesis, since the growth factor signaling mechanisms of embryogenesis become homeostatic mechanisms in the offspring [47]. As such, they also can discriminate between adaptive and maladaptive genetic mutations that affect homeostasis, either indirectly through the developmental process, or directly through the regulatory mechanisms of physiology.

## 8. Diachronic Growth Factor Signaling Mechanisms are Common to Development, Homeostasis and Regeneration

Embryonic growth and development are determined by paracrine growth factor-receptor signaling, forming the spatio-temporal patterns that provide the form and function of tissues and organs [48]. The lung is the most extensively characterized organ because of its critical importance to survival at the time of birth in humans. 

In order to generate an efficient diffusible surface for gas exchange, the lung endoderm grows and differentiates to form the conducting airways and alveoli interfaced with a complementary vascular system. The key genes involved in lung development are highly conserved across phylogeny at least as far back as the swim bladder of physostomous fish [49]. Signaling by the Insulin-like Growth Factor, Epidermal Growth Factor and Transforming Growth Factorβ/Bone Morphogenetic Protein pathways, and extracellular matrix components and integrins also directs lung morphogenesis. The soluble growth factors secreted by lung mesoderm are a complete inducer of lung morphogenesis [48], first observed by Clifford Grobstein [50].

## 9. Homeostasis as the Agent for Change During the Vertebrate Water-Land Transition-Emergence

Duplication of specific genes is well known to have occurred during the vertebrate water-land transition. In fish, the Parathyroid Hormone-related Protein Receptor (PTHrPR) [6], the βAdrenergic Receptor (βARs) [7] and the Glucocorticoid Receptor (GCRs) [8] all mediate physiologic functions for adaptation to water. Some 250–500 million years ago increasing carbon dioxide in the atmosphere [51] generated a ‘green house’ effect, causing the ambient temperature to rise, drying up oceans, lakes and rivers [52]. The existential vertebrate transition from water to land was accommodated by the duplication of these genes (PTHrPR, βAR, GR), which were repurposed for respiratory [53], skeletal [54], renal [55] and dermal [56] functions. The amplification of these specific genes was not merely fortuitous, as the literature would have us believe [57]; they were essential for either adapting to land or becoming extinct [58,59]. Why they duplicated is answered by thinking of them in the physiologic contexts of their functions in lung breathing, neuroendocrine stress, and metabolism. How these genetic changes occurred is speculative, but would have occurred as a result of microvascular shear stress causing remodeling of these specific tissues and organs, as follows.

## 10. Homeostasis as the Consequence of Developmental Mechanisms

**Figure 3:**
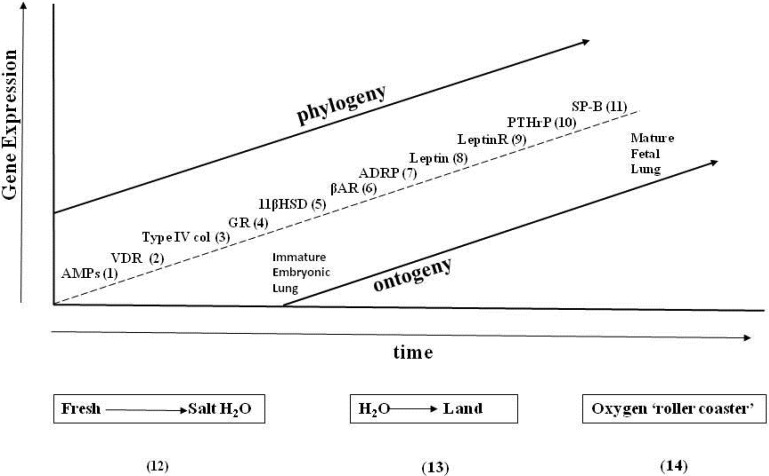
Extrinsic and intrinsic selection pressures for the genes of lung phylogeny and ontogeny. The effects of the extrinsic factors (salinity, land nutrients, and oxygen on the x-axis) on genes that determine the phylogeny and ontogeny of the mammalian lung alternate sequentially with the intrinsic genetic factors (y-axis), highlighted by the squares and circles, respectively. Steps 1–11 appear in the sequence they appear during phylogeny and ontogeny: (1) AMPs; (2) VDR; (3) type IV collagen; (4) GR; (5) 11β HSD; (6) βAR; (7) ADRP; (8) leptin; (9) leptin receptor; (10) PTHrP; and (11) SP-B. Steps 12–14 are major geologic epochs that have “driven” intrinsic lung evolution.

As one reads the Evolutionary Biology literature, observations of pre-adaptations, or exaptations, come up recurrently. Perhaps that is an artifactual consequence of looking at the process of evolution from its ends instead of its means. A priori, if one follows pre-adaptation to its logical extension, it would terminate in the unicellular state, which is the origin of metazoans, using ontogenetic and phylogenetic principles. However, moving in a prograde direction, by thinking about the evolutionary adaptations in the context of the ever-changing environment, the causal relationships become clear, as has previously been shown for the evolution of the lung [10]—by regressing the genes that have determined structure and function during lung ontogeny and phylogeny against major epochs in the environment-ocean salinity, the drying-up of the oceans, fluctuations in atmospheric oxygen-expressed as Cartesian Coordinates (Figure 3), one can see the adaptive mechanisms of internal selection due to physical forces, mediated by physiologic stress, starting with the advent of the peroxisome as balancing selection for calcium dyshomeostasis [60]. The lung is the optimal example, or cipher, for such evolutionary changes in vertebrate visceral physiology because of the powerful positive selection pressure for its evolution during the water-land transition—there were no alternatives, it was either adapt for air breathing or die.

The lesson learned from that event becomes even more self-evident when thinking about the specific implications of the three gene duplications that occurred during that transition (see Figure 3)—the GR (step 4), the βAR (step 6), and the PTHrP Receptor (PTHrPR) (step 10) may have duplicated primarily because PTHrP promotes bone remodeling, since the vertebrate skeleton is known to have evolved at least five times based on the fossil record [61], providing ample opportunity for the coevolution of the visceral organs necessary for land adaptation. But PTHrP signaling is also important for air breathing and for the skin as a barrier [56], both of which were also necessary for terrestrial adaptation. Experimentally, if you delete the PTHrP gene from a developing mouse, it causes developmental deficits in the lung (no alveoli), bone (failure to calcify), and skin (immature barrier) [56], consistent with all of the aforementioned phenotypes.

The literature would lead us think that these gene duplications occurred by chance alone [57], but these genetic mutations are not considered within their ecologic and biologic contexts. The physiologic stresses incurred by the transitioning from water to land-air breathing, increased gravitational force, loss of water and electrolytes- were enormous. Vascular shear would have been greatest within those specific microvascular beds on which that transition was dependent-lung, bone, kidney-generating Radical Oxygen Species known to cause gene mutations and duplications in the process [62]. Over time, such mutations would have formed structures and functions for adaptation, and secondarily affected positive selection for the molecular traits involved, or the organism would have become extinct—what Darwinian evolutionists refer to metaphorically as ‘Survival of the Fittest’, though now with specific, causally-testable hypotheses.

**Figure 4:**
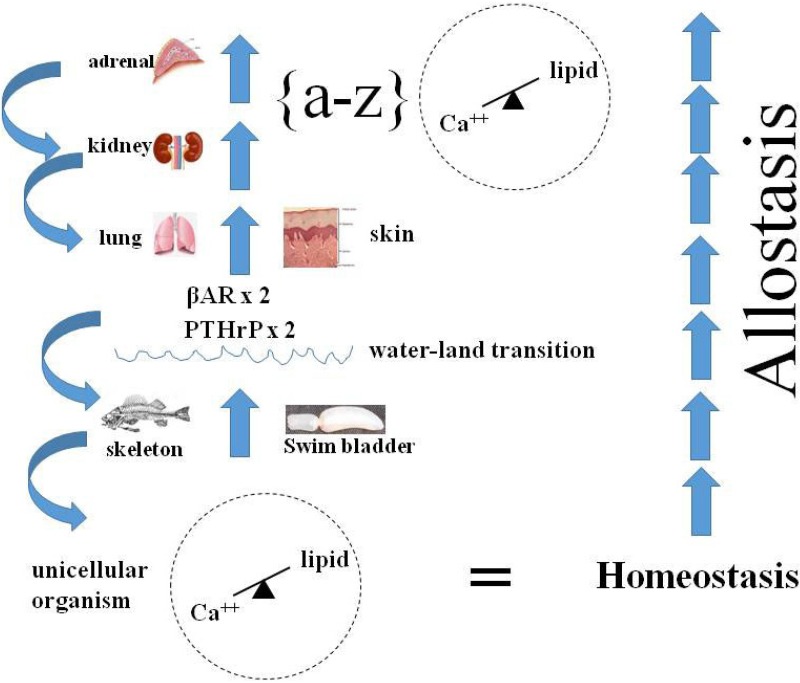
Physiologic Adaptation. The ontogenetic and phylogenetic integration (**∫**) of calcium-lipid homeostasis, from unicellular organism incorporation of lipid into the plasma lemma to multicellular organism calcium/lipid epistatic homeostasis fostered the Evolution of metazoans. This figure, focuses on the specific stress of the water-land transition on the Evolution of a wide variety of organs-bone, lung, skin, kidney, adrenal-resulting from the duplication of the PTHrP Receptor gene in fish, followed by the βAdrenergic Receptor (βAR) gene, culminating in integrated physiology, or allostasis (on far right). Internal selection was mediated through selection pressure on homeostatic mechanisms mediated by paracrine cell-cell interactions; as vertebrates adapted to land, the PTHrP signaling mechanism iteratively allowed for physiologic adaptations to air breathing (skin, lung), prevention of dessication (skin, kidney) and ‘fight or flight’ (adrenal). The blue arrows on the far left signify how evolved traits refer back to their antecedents, or are exapted.

Genetic remodeling of the alveolar bed for stretch-regulated PTHrP signaling would have had dual physiologic adaptational advantages (Figure 4), initially by stimulating alveolar surfactant production [63], relieving the inevitable episodic stress of alveolar insufficiency, resulting in hypoxia during the process of evolution. That would have been followed by PTHrP acting both to generate more alveoli [63], and as a potent vasodilator [64] accommodating the concomitant increase in alveolar microvascular blood flow.

Another gene duplication that occurred during the vertebrate water-land transition was for the beta adrenergic receptor (βAR), which ultimately provided for the local pulmonary regulation of alveolar capillary blood pressure, necessitated by the constraints of the systemic blood pressure imposed on the alveolar capillary system [65]. That new physiologic trait may have evolved as a result of the coevolution of PTHrP signaling in the anterior pituitary [66], and in the adrenal cortex [67], increasing ACTH and glucocorticoid production, respectively, in adaptation to terrestrial physiologic stress. The resultant increased responsiveness to physiologic stress by the Pituitary-Adrenal Axis (PAA) would have amplified adrenalin production, since the corticoids produced in the adrenal cortex pass through the adrenal medulla, where they physiologically stimulate the rate-limiting step in adrenalin production, Catechol-O-Methyltransferase, or COMT [68]. Moreover, the increased PTHrP flowing through the medulla may actually have promoted the formation of more vascular arcades in the mammalian adrenal medulla [69], since PTHrP is angiogenic [70]. Terrestrial adaptation mediated by the PAA may have been brought on by the episodic pulmonary insufficiencies that inevitably would have occurred during the step-wise morphogenetic processes of lung evolution in adaptation to land, causing intermittent periods of hypoxia, the most potent physiologic agonist for the stress reaction.

Furthermore, that adaptation to hypoxia would have been reinforced by the concomitant fluctuations in atmospheric oxygen over the course of the last 500 million years [51], ranging between 15% and 35% during the Phanerozoic period. The resultant over-expression of adrenalin would have transiently alleviated the constraint on the alveolar walls by increasing surfactant secretion [71], lowering alveolar surface tension, in combination with transiently increased blood flow due to PTHrP’s potent vasodilatory effect [64]. It should be emphasized that all of these pre-adapted physiologic traits were recruited in service to optimized air breathing, and ultimately were selected for by the concerted effects of internal selection and natural selection.

Ultimately, the well-recognized phylogenetic increase in alveolar capillary βAR density, hypothetically due to the stress-stimulated effect of glucocorticoids on βAR expression, alleviated the constraint on the circulatory system by allowing for the independent regulation of the pulmonary and systemic blood pressures [65], accommodating the ever-increasing evolutionary metabolic demand for the increase in lung surface area to facilitate gas exchange based on this allostatic mechanism [20,31,32].

Interestingly, the glucocorticoid receptor also evolved from the mineralocorticoid receptor during this same window of time due to two gene mutations [8]. The evolution of glucocorticoid signaling from the mineralocorticoid signaling mechanism would have relieved the maladaptational elevation in blood pressure caused by increased gravitational effects of land habitation, which was also constraining the evolution of the lung surface area, as indicated above—a ‘two-hit’ hypothesis; the concomitant positive selection for glucocorticoids as agonists for βAR expression [68] would have synergized with the evolution of the local alveolar blood pressure regulation in service to increased oxygenation.

In support of the relevance of βARs to land adaptation, disruption of βAR signaling during embryonic mouse development inhibits normal heart development [72]. This should not be surprising, since the expanding surface area of the lung evolved in tandem with the heart, from the one-chambered Annelid worm heart, to the two-chambered fish heart, to the three-chambered frog heart, to the four-chambered mammalian heart [73]. The coordinated evolution of the lung and heart by the PTHrPR and βAR gene duplications through the complimentary interactions described above would have facilitated land habitation and further evolutionary adaptation in response to tandem increases in both gas-exchange and blood pressure regulation under physiologic stress conditions.

Moreover, this synergistic relationship between PTHrP signaling and βAR signaling may have fostered the formation of glomeruli in the phylogenetic transition of the kidney glomerulus from the primitive capillary system, or glomus of the fish kidney [74] to the glomerulus of the amphibian, reptilian, mammalian and avian kidneys. Such remodeling culminates in homeostatic PTHrP regulation of the mesangium controlling glomerular filtration [55], and βARs regulating urinary output under stress [75].

## 11. PTHrP and Hypothalamic-Pituitary-Adrenal Regulation of Physiologic Stress

Extensive experimental evidence from our laboratory has shown the central role of PTHrP in normal lung development [3], beginning with the embryonic mouse knockout for PTHrP causing impaired lung development due to failure to form alveoli [53]. In various insult models for lung disease-oxotrauma, barotrauma, infection—we have documented the decrease in PTHrP expression in all of these instances [76]. Moreover, infants who develop Bronchopulmonary Dysplasia are PTHrP deficient based on measurement of the molecule in bronchoalveolar lavage [77].

## 12. The Role of PTHrP Expression in Adrenal Corticoid Synthesis

More recently, it has been discovered that PTHrP is expressed in the pituitary [66], where it stimulates ACTH, and in the adrenal cortex [67] where it mediates the ACTH stimulation of corticoid synthesis. As a consequence, this pathway would have amplified the physiologic stress response by increasing adrenalin production by the adrenal medulla [78]. This mechanism may have evolved during the water-land transition, wherein the lung would periodically have been unable to generate adequate amounts of oxygen, causing hypoxia. Hypoxia is the most potent physiologic agonist known; by stimulating adrenalin production, which stimulates surfactant secretion, it would have transiently alleviated the atelectatic stress on the lung [71].

Such a mechanism may refer all the way back to the *Cenozoic era*, when our common rodent-like ancestor had to be nimble in order to avoid being crushed or eaten by predators. Over time, the collective stress on the microvasculature of the lung, pituitary and adrenal cortex may have ‘remodelled’ all of these structures, including the adrenal medulla. The latter is known to have evolved a complex vascular arcade in mammals [69], acting like an ‘echo chamber’ to enhance adrenalin production in response to stress, both baseline and regulated states.

## 13. Homeostatic Regulation is Diachronic

The key to understanding the interrelationship between homeostasis and embryogenesis lies in recognizing the diachronic nature of the overall mechanism of evolution (see Figure 2). During the process of embryogenesis, growth factor signaling determines the structure and function of the offspring. Subsequently, the homeostatic set-point is challenged during postnatal life, overtly being maintained by many of the same signaling principles used for embryogenesis. If the limits of homeostasis are challenged, growth factor signaling mechanisms may revert to their ancestral form in order to sustain the organism, sometimes causing fibrosis as the structural default mode that grants the organism the ability to reproduce under suboptimal physiologic conditions. Under extreme conditions, such as mass extinctions [79], or the water-land transition [52], physiologic stress has caused pragmatic remodeling of organs in order to adapt [2,3,4]. Those members of the species best suited to mount such an adaptive strategy pass such homeostatically adaptive genes on to their offspring, generating a heritable phenotype in the process. Hence, the relationship between homeostasis and phenotypic change is a continuum, mediated by growth factor signaling properties that are mechanistically common to both.

This intimate relationship between environmental physiologic stress, homeostasis and remodeling goes all the way back to the inception of life, protocells forming from micelles, generating an internal environment (*milieu interieur*) using both the cell membrane and the endomembrane systems to compartmentalize physiologic properties; the resulting generation of negentropy, sustained by chemiosmosis [3] forms the basis for the vital principle. The ability to recapitulate this process from one generation to the next, acquiring new ‘knowledge’ through reproduction and epigenetics allows the system to perpetuate itself indefinitely, or become extinct.

## 14. Allostasis as Integrated Homeostasis

The concept of allostasis is yet another example of how our seeming inability to see homeostasis in its fullest form, diachronically, as the mechanism underlying all of biology, has distracted us from seeing the continuum of biologic processes. McEwen and Wingfield [31] define allostasis as a process that supports homeostasis, the ‘set-points’ and other boundaries of control that must change, the physiological and/or life history stages that must change to achieve stability. Allostasis clarifies an inherent ambiguity in the term ‘homeostasis’ and distinguishes between the systems that are essential for life (“homeostasis”) and those that maintain these systems in balance (“allostasis”) as environment and life history stage changes.

This perception of a supra-homeostatic control system independent of homeostasis is the consequence of failing to recognize the primacy of the cell in mediating the evolutionary principle yet again. If in effect life is a continuum that emanates from the unicellular state, then homeostasis functions at all levels of biology as a fractal, independent of scale [4]. So the properties of allostasis are a higher-level expression of the same homeostatic principles expressed at the cellular, tissue and organ levels. The examples used by McEwen and Wingfield [31,32]—blood pressure, metabolism, pH, complex patterns of bird migration—are all derived from homeostatic regulation of the unicellular state, having evolved in support of multicellular organisms. Migratory birds were used by Ernst Mayr [80] to exemplify the difference between proximate and ultimate causation in evolutionary biology, which drove a wedge between those interested in structure-function relationships (proximate) and the process of evolution itself (ultimate), generating volumes of descriptive data, undermining any attempt to understand how and why evolution has occurred based on principles of cell biology [4].

Elsewhere [2,3,4], the life span of the organism as a continuous series of ligand-receptor interactions from morphogenesis to the maintenance of physiologic homeostasis, to the loss of homeostatic control mechanisms during aging, culminating in death has been formulated. Seen in this light, allostasis takes on a very different set of characteristics, stress having short-term effects that are physiologically beneficial for the reproductive strategy; but over the long haul, such adaptive responses can have deleterious effects that occur as unintended consequences of the optimization of the primary homeostatic mechanisms involved. In other words, acceleration of development would bring on precocious aging and death as a continuous mechanism selecting for the unicellular state. By conventionally focusing on the pathological aspects of allostatic load, we get a skewed view of its function. For example, we have yet to understand the role of premature adrenarche—the production of androgens by the adrenal cortex in the onset of puberty. We know that it is associated with Intrauterine Growth Retardation (IUGR) [81] and overweight children [82], particularly as it relates experimentally to altered metabolism *in utero* [83]. Yet, from a cellular mechanistic perspective, precocious puberty due to low food abundance during development *in utero* is adaptive, accelerating sexual maturation in the offspring, advancing the option for the next generation to live in an environment of greater food abundance. This is consistent with the fact that IUGR is associated with precocious adrenarche, known to cause early puberty [81]. So these are diametrically opposite perspectives on the significance of homeostasis during the life history of the organism.

Furthermore, McEwen and Wingfield [31,32] characterize allostasis as ‘protection *vs.* damage’, yet again only seeing the immediate consequences of metabolic control on physiology when the true agenda—the acquisition of newly-acquired epigenetic mutations—is actually of an intergenerational nature, providing a very different interpretation of the observed phenomenon. As cited above, Waddington [30] intimated that we have to see the organism as both its past and its future, not just as it appears in its current condition in order to understand epigenetic inheritance. Here again, the interpretation of protection *vs*. damage in the context of adaptive and maladaptive is very different from that perspective—even what appears as damage is actually the consequence of an adaptive response from the perspective of opitimized reproduction.

## 15. Conclusions

Our ignorance of the fundamental First Principles of Physiology have led us astray. Conversely, by focusing on the unicellular state as the primary level of selection, we gain insight to such ontologic and epistemologic principles as the life cycle, embryogenesis, life history and homeostasis. Such deep understanding is of critical importance to our effective utilization of genomic information.

Both Duhem [84] and W.O.V. Quine [85] have pointed out the limitations of science as ‘underdetermination’. Quine said that knowledge is “a man-made fabric which impinges on experience only along the edges”, and then goes on to say that “a conflict with experience at the periphery occasions readjustments in the interior of the field”. If we knew what our physiologic ‘experience’ actually constituted, perhaps we could avoid the pitfalls of such scientific subjectivity.

## References

[1] Birks J.B. (1962). Rutherford at Manchester.

[2] Torday J.S., Rehan V.K. (2012). Evolutionary Biology, Cell-Cell Signaling and Complex Disease.

[3] Torday J.S. (2013). Evolutionary biology redux. Perspect. Biol. Med..

[4] Torday J.S. (2015). A central theory of biology. Med. Hypotheses.

[5] Jacob F. (1977). Evolution and tinkering. Science.

[6] Pinheiro P.L., Cardoso J.C., Power D.M., Canário A.V. (2012). Functional characterization and evolution of PTH/PTHrP receptors: Insights from the chicken. BMC Evol. Biol..

[7] Aris-Brosou S., Chen X., Perry S.F., Moon T.W. (2009). Timing of the functional diversification of alpha- and beta-adrenoceptors in fish and other vertebrates. Ann. N. Y. Acad. Sci..

[8] Bridgham J.T., Carroll S.M., Thornton J.W. (2006). Evolution of hormone-receptor complexity by molecular exploitation. Science.

[9] Lane N., Allen J.F., Martin W. (2010). How did LUCA make a living? Chemiosmosis in the origin of life. Bioessays.

[10] Lane N., Martin W.F. (2012). The origin of membrane bioenergetics. Cell.

[11] Martin W.F., Sousa F.L., Lane N. (2014). Energy at life’s origin. Science.

[12] Bhagavan N.V. (2002). Medical Biochemistry.

[13] Reydon T.A. (2006). Generalizations and kinds in natural science: The case of species. Stud. Hist. Philos. Biol. Biomed. Sci..

[14] Pennazio S. (2009). Homeostasis: A history of biology. Riv. Biol..

[15] Roux E. (2014). The concept of function in modern physiology. J. Physiol..

[16] MacDougall-Shackleton S.A. (2011). The levels of analysis revisited. Philos. Trans. R. Soc. Lond. B.

[17] Lwebuga-Mukasa J.S., Ingbar D.H., Madri J.A. (1986). Repopulation of a human alveolar matrix by adult rat type II pneumocytes *in vitro*. A novel system for type II pneumocyte culture. Exp. Cell Res..

[18] Torday J.S., Rehan V.K. (2006). Up-regulation of fetal rat lung parathyroid hormone-related protein gene regulatory network down-regulates the Sonic Hedgehog/Wnt/betacatenin gene regulatory network. Pediatr. Res..

[19] Gleason C.A., Devaskar S. (2011). Avery’s Diseases of the Newborn.

[20] Torday J.S., Rehan V.K., Hicks J.W., Wang T., Maina J., Weibel E.R., Hsia C.C., Sommer R.J., Perry S.F. (2007). Deconvoluting lung evolution: From phenotypes to gene regulatory networks. Integr. Comp. Biol..

[21] Sahni R., Polin R.A. (2013). Physiologic underpinnings for clinical problems in moderately preterm and late preterm infants. Clin. Perinatol..

[22] Chilosi M., Poletti V., Zamò A., Lestani M., Montagna L., Piccoli P., Pedron S., Bertaso M., Scarpa A., Murer B. (2003). Aberrant Wnt/β-catenin pathway activation in idiopathic pulmonary fibrosis. Am. J. Pathol..

[23] Marieb E.N., Hoehn K. (2007). Human Anatomy & Physiology.

[24] Besnard V., Wert S.E., Stahlman M.T., Postle A.D., Xu Y., Ikegami M., Whitsett J.A. (2009). Deletion of Scap in alveolar type II cells influences lung lipid homeostasis and identifies a compensatory role for pulmonary lipofibroblasts. J. Biol. Chem..

[25] Torday J.S., Powell F.L., Farmer C.G., Orgeig S., Nielsen H.C., Hall A.J. (2010). Leptin integrates vertebrate evolution: From oxygen to the blood-gas barrier. Respir. Physiol. Neurobiol..

[26] Torday J.S., Rehan V.K. (2004). Deconvoluting lung evolution using functional/comparative genomics. Am. J. Respir. Cell Mol. Biol..

[27] Reid R.G.B. (1985). Evolutionary Biology: The Unfinished Synthesis.

[28] Bernard C. (1865). Introduction à L’étude de la Médecine Expérimentale.

[29] Cannon W.B. (1929). Organization for physiological homeostasis. Physiol. Rev..

[30] Waddington C.H. (1957). The Strategy of the Genes.

[31] McEwen B.S., Wingfield J.C. (2003). The concept of allostasis in biology and biomedicine. Horm. Behav..

[32] McEwen B.S., Wingfield J.C. (2010). What is in a name? Integrating homeostasis, allostasis and stress. Horm. Behav..

[33] Rué P., Arias A.M. (2015). Cell dynamics and gene expression control in tissue homeostasis and development. Mol. Syst. Biol..

[34] Kotas M.E., Medzhitov R. (2015). Homeostasis, inflammation, and disease susceptibility. Cell.

[35] Mayer E.A. (2011). Gut feelings: The emerging biology of gut-brain communication. Nat. Rev. Neurosci..

[36] Kirschner M., Gerhart J., Mitchison T. (2000). Molecular “vitalism”. Cell.

[37] Fankhauser G. (1945). Maintenance of normal structure in heteroploid salamander larvae, through compensation of changes in cell size by adjustment in cell number and cell shape. J. Exp. Zool..

[38] Fankhauser G. (1972). Memories of great embryologists. Am. Sci..

[39] Campbell D.T. (1974). Studies in the Philosophy of Biology: Reduction and Related Problems.

[40] Noble D. (2012). A theory of biological relativity: No privileged level of causation. Interface Focus.

[41] Rehan V.K., Liu J., Naeem E., Tian J., Sakurai R., Kwong K., Akbari O., Torday J.S. (2012). Perinatal nicotine exposure induces asthma in second generation offspring. BMC Med..

[42] Waterland R.A., Jirtle R.L. (2004). Early nutrition, epigenetic changes at transposons and imprinted genes, and enhanced susceptibility to adult chronic diseases. Nutrition.

[43] Jaenisch R., Bird A. (2003). Epigenetic regulation of gene expression: How the genome integrates intrinsic and environmental signals. Nat. Genet..

[44] Cropley J.E., Suter C.M., Beckman K.B., Martin D.I. (2006). Germ-line epigenetic modification of the murine Avy allele by nutritional supplementation. Proc. Natl. Acad. Sci. USA.

[45] Rehan V.K., Liu J., Sakurai R., Torday J.S. (2013). Perinatal nicotine-induced transgenerational asthma. Am. J. Physiol. Lung Cell Mol. Physiol..

[46] Darwin C. (1859). On the Origin of Species.

[47] Calkins K., Devaskar S.U. (2011). Fetal origins of adult disease. Curr. Probl. Pediatr. Adolesc. Health Care.

[48] Warburton D., Schwarz M., Tefft D., Flores-Delgado G., Anderson K.D., Cardoso W.V. (2000). The molecular basis of lung morphogenesis. Mech. Dev..

[49] Zheng W., Wang Z., Collins J.E., Andrews R.M., Stemple D., Gong Z. (2011). Comparative transcriptome analyses indicate molecular homology of zebrafish swimbladder and mammalian lung. PLoS ONE.

[50] Grobstein C. (1967). Mechanisms of organogenetic tissue interaction. Natl. Cancer Inst. Monogr..

[51] Berner R.A., Vandenbrooks J.M., Ward P.D. (2007). Evolution. Oxygen and evolution. Science.

[52] Romer A.S. (1949). The Vertebrate Story.

[53] Rubin L.P., Kovacs C.S., de Paepe M.E., Tsai S.W., Torday J.S., Kronenberg H.M. (2004). Arrested pulmonary alveolar cytodifferentiation and defective surfactant synthesis in mice missing the gene for parathyroid hormone-related protein. Dev. Dyn..

[54] Kronenberg H.M., Karaplis A.C., Lanske B. (1996). Role of parathyroid hormone-related protein in skeletal development. Ann. N. Y. Acad. Sci..

[55] Bosch R.J., Rodríguez-Puyol D., Bover J., Rodríguez-Puyol M. (1999). Parathyroid hormone-related protein: Roles in the glomerulus. Exp. Nephrol..

[56] Karaplis A.C., Kronenberg H.M. (1996). Physiological roles for parathyroid hormone-related protein: Lessons from gene knockout mice. Vitam. Horm..

[57] Weissmann G. (2007). Evo-Devo and the lungfish: The last gasp of intelligent design. FASEB J..

[58] Torday J.S., Rehan V.K. (2007). The evolutionary continuum from lung development to homeostasis and repair. Am. J. Physiol. Lung Cell Mol. Physiol..

[59] Torday J.S., Rehan V.K. (2011). A cell-molecular approach predicts vertebrate evolution. Mol. Biol. Evol..

[60] De Duve C. (1969). Evolution of the peroxisome. Ann. N. Y. Acad. Sci..

[61] Clack J.A. (2002). Gaining Ground.

[62] Storr S.J., Woolston C.M., Zhang Y., Martin S.G. (2013). Redox environment, free radical, and oxidative DNA damage. Antioxid. Redox Signal..

[63] Rubin L.P., Kifor O., Hua J., Brown E.M., Torday J.S. (1994). Parathyroid hormone (PTH) and PTH-related protein stimulate surfactant phospholipid synthesis in rat fetal lung, apparently by a mesenchymal-epithelial mechanism. Biochim. Biophys. Acta.

[64] Gao Y., Raj J.U. (2005). Parathyroid hormone-related protein-mediated responses in pulmonary arteries and veins of newborn lambs. Am. J. Physiol. Lung Cell Mol. Physiol..

[65] West J.B., Mathieu-Costello O. (1999). Structure, strength, failure, and remodeling of the pulmonary blood-gas barrier. Annu. Rev. Physiol..

[66] Kawashima M., Takahashi T., Yanai H., Ogawa H., Yasuoka T. (2005). Direct action of parathyroid hormone-related peptide to enhance corticosterone production stimulated by adrenocorticotropic hormone in adrenocortical cells of hens. Poult. Sci..

[67] Nakayama H., Takahashi T., Oomatsu Y., Nakagawa-Mizuyachi K., Kawashima M. (2011). Parathyroid hormone-related peptide directly increases adrenocorticotropic hormone secretion from the anterior pituitary in hens. Poult. Sci..

[68] Kvetnansky R., Lu X., Ziegler M.G. (2013). Stress-triggered changes in peripheral catecholaminergic systems. Adv. Pharmacol..

[69] Wurtman R.J. (2002). Stress and the adrenocortical control of epinephrine synthesis. Metabolism.

[70] Park S.I., Lee C., Sadler W.D., Koh A.J., Jones J., Seo J.W., Soki F.N., Cho S.W., Daignault S.D., McCauley L.K. (2013). Parathyroid hormone-related protein drives a CD11b+Gr1+ cell-mediated positive feedback loop to support prostate cancer growth. Cancer Res..

[71] Lawson E.E., Brown E.R., Torday J.S., Madansky D.L., Taeusch H.W. (1978). The effect of epinephrine on tracheal fluid flow and surfactant efflux in fetal sheep. Am. Rev. Respir. Dis..

[72] Rohrer D.K., Desai K.H., Jasper J.R., Stevens M.E., Regula D.P., Barsh G.S., Bernstein D., Kobilka B.K. (1996). Targeted disruption of the mouse beta1-adrenergic receptor gene: Developmental and cardiovascular effects. Proc. Natl. Acad. Sci. USA.

[73] Simões-Costa M.S., Vasconcelos M., Sampaio A.C., Cravo R.M., Linhares V.L., Hochgreb T., Yan C.Y., Davidson B., Xavier-Neto J. (2005). The evolutionary origin of cardiac chambers. Dev. Biol..

[74] Smith H.W. (1953). From Fish to Philosopher.

[75] Hart P.D., Bakris G.L. (2007). Should beta-blockers be used to control hypertension in people with chronic kidney disease?. Semin. Nephrol..

[76] Cerny L., Torday J.S., Rehan V.K. (2008). Prevention and treatment of bronchopulmonary dysplasia: Contemporary status and future outlook. Lung.

[77] Rehan V.K., Torday J.S. (2006). Lower parathyroid hormone-related protein content of tracheal aspirates in very low birth weight infants who develop bronchopulmonary dysplasia. Pediatr. Res..

[78] Wong D.L. (2006). Epinephrine biosynthesis: Hormonal and neural control during stress. Cell Mol. Neurobiol..

[79] Kolbert E. (2014). The Sixth Extinction.

[80] Mayr E. (1961). Cause and effect in biology. Science.

[81] Longo S., Bollani L., Decembrino L., di Comite A., Angelini M., Stronati M. (2013). Short-term and long-term sequelae in intrauterine growth retardation (IUGR). J. Matern. Fetal Neonatal. Med..

[82] Salam R.A., Das J.K., Bhutta Z.A. (2014). Impact of intrauterine growth restriction on long-term health. Curr. Opin. Clin. Nutr. Metab. Care.

[83] Ross M.G., Desai M. (2013). Developmental programming of offspring obesity, adipogenesis, and appetite. Clin. Obstet. Gynecol..

[84] Duhem P., Wiener P.P. (1954). The Aim and Structure of Physical Theory.

[85] Quine W.O.V. (2015). Word and Object.

